# Latent Transforming Growth Factor β-Binding Protein 4 Is Downregulated in Esophageal Cancer via Promoter Methylation

**DOI:** 10.1371/journal.pone.0065614

**Published:** 2013-05-31

**Authors:** Insa Bultmann, Anne Conradi, Celine Kretschmer, Anja Sterner-Kock

**Affiliations:** 1 Center for Experimental Medicine, Medical Faculty, University of Cologne, Cologne, Germany; 2 Med. Klinik Hepatologie und Gastroenterologie, Charité Berlin, Berlin, Germany; University of Saarland Medical School, Germany

## Abstract

Latent transforming growth factor β-binding protein 4 (LTBP4) is an extracellular matrix molecule that is a member of important connective tissue networks and is needed for the correct folding and the secretion of TGF-β1. LTBP4 is downregulated in carcinomas of various tissues. Here we show that LTBP4 is also downregulated in adenocarcinomas and squamous cell carcinomas of the esophagus *in vitro* and *in vivo*. Re-expression of LTBP4 in esophageal cancer cell lines reduced cell migration ability, whereas cell viability and cell proliferation remained unchanged. Hypermethylation of the promoter regions of the two main human LTBP4 transcriptional forms, LTBP4L and LTBP4S, was found to be involved in LTBP4 silencing. Detailed investigations of the methylation patterns of the promoter regions of LTBP4L and LTBP4S identified GATA1, SP1, E2F4 and SMAD3 as potential transcription factors involved in LTBP4 expression. In *in vitro* transcription factor activity studies we discovered E2F4 as novel powerful regulator for LTBP4S expression.

## Introduction

In 2008 esophageal cancer was with estimated 482,000 new cases the eighth most common malignancy in the world and with 407,000 deaths the sixth most common cause of death [1]. In the United States of America the median age of diagnosis of esophageal cancer is 68 years and about 80% of all new cases are diagnosed in men. The 5-year overall survival rate increased from 5% in 1975 to 19 % in 2001 [2]. In Europe the mean age of diagnosis is 70 years and 67% of newly diagnosed cases are males. The 5-year overall survival rate is with 9.8% much lower than in the United States of America [3].

The two predominant histological types of esophageal cancer are adenocarcinomas (EAC) and squamous cell carcinomas (ESCC). Both apparently differ in their patterns of incidence and have an own distinct etiology [4]. In Europe the 1-year relative survival of patients with ESCC is 33.9% and 37.9% of EAC-patients, however, the 5-year relative survival is with 10.1% and 10.6%, respectively, almost identical [3]. One established risk factor for EAC and ESSC is cigarette smoking, while alcohol consumption is assumed as risk factor only for ESCC [4]. Whereas, obesity, gastroesophageal reflux disease and Barretts’s esophagus are only risk factors for EAC [4]. The daily intake of fresh fruits and vegetables is associated with decreased risk of both histological types of esophageal cancer [4].

Changes in the extracellular matrix play an important role in the carcinogenesis of esophageal cancer. Degradation and reduced expression of extracellular matrix proteins are related to tumor progression, including invasion and migratory potential, as well as metastasis, cell proliferation and angiogenesis [5].

The extracellular matrix protein latent transforming growth factor β-binding protein 4 (LTBP4) is one of four isoforms of LTBPs (LTBP1–4) and belongs to the fibrillin/LTBP family of glycoproteins. LTBP1, LTBP3 and LTBP4 bind covalently to transforming growth factor βs (TGF-βs) and are needed for the correct folding and the secretion of TGF-βs [6], [7]. LTBP4 only binds TGF-β1, whereas LTBP1 and 3 bind all three TGF-β isoforms [7].

TGF-β1 is a pluripotent and omnipresent cytokine belonging to a superfamily of structurally related growth factors with well documented roles in cellular proliferation, differentiation, apoptosis, adhesion, and extracellular matrix deposition [8], [9].

In addition to being crucial for chaperoning and transporting TGF-β outside the cell, LTBPs have been shown to be associated members of connective tissue networks such as the microfibril/elastic fiber network and the fibronectin network [10]. The incorporation of LTBPs into the ECM is crucial for regulation of latent TGF-β storage and activation [11].

Various transcriptional forms of LTBP4 exist in humans and the main forms are a long (LTBP4L; NM_001042544) and a short (LTBP4S; NM_001042545) form. Using two independent promoters both transcriptional forms are expressed in different expression patterns in a tissue-specific manner [12].

It has been shown that dysregulated expression of LTBP isoforms is related to the onset of epithelial neoplasms [13], [14], [15], [16], [17], [18]. LTBP1 is downregulated in a variety of human epithelial neoplasms of liver, ovaries and neuroendocrine tumors of the digestive system [14], [16], [18]. LTBP2 is downregulated in ESCC and nasopharyngeal carcinomas [13], [15]. LTBP4 is downregulated in human and murine ductal carcinoma *in situ* and mammary adenocarcinomas [17].

The mechanisms which lead to downregulation of LTBPs in various epithelial neoplasms are largely unknown. However, for LTBP2 it is known that hypermethylation of the promoter is responsible for the downregulation in ESCC and nasopharyngeal carcinomas [13], [15].

The present study investigates the protein expression of the extracellular matrix protein LTBP4 in different stages of esophageal cancer progression, as well as in various adenocarcinomas of the gastrointestinal tract. Furthermore, we investigated the potential regulatory mechanism responsible for LTBP4 inactivation in esophageal cancer.

## Results

### LTBP4 is downregulated in different stages of esophageal cancer progression

In the present study we investigated the protein expression of LTBP4 in gastrointestinal carcinomas and in particular in neoplasias and preneoplasias of the esophagus. Therefore, we first assessed the expression of LTBP4 levels in different cancer tissue microarrays by immunohistochemical staining.

**Figure 1:**
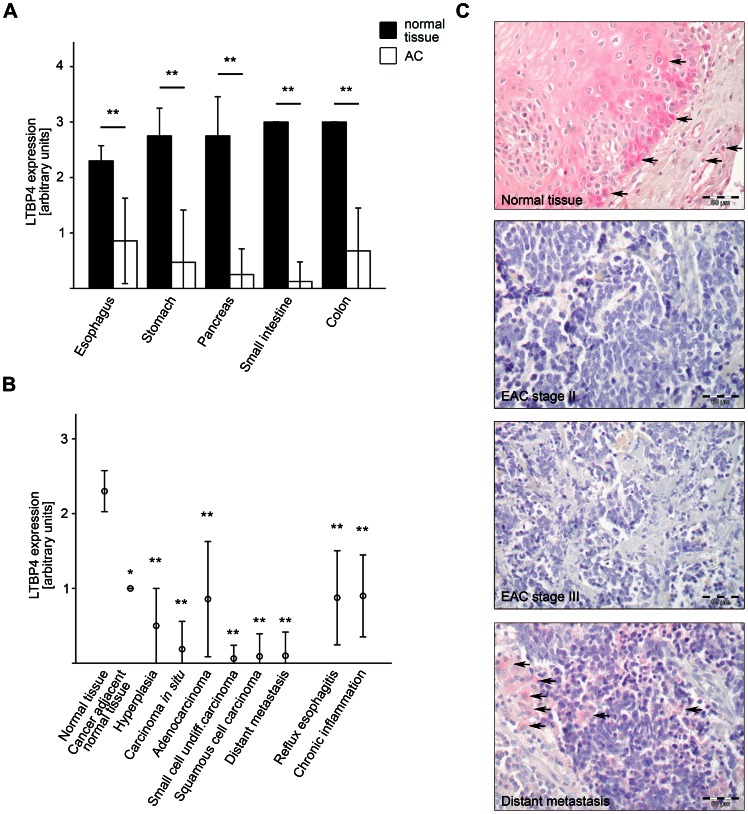
LTBP4 is downregulated in gastrointestinal cancer. A) LTBP4 protein expression levels were assessed by immunohistochemistry in tissue microarrays of patient adenocarcinomas (AC) of esophagus, stomach, pancreas, small intestine and colon compared to normal tissue of the same organ. B) LTBP4 protein expression levels were assessed by immunohistochemistry in patient tissues of esophageal cancer progression compared to normal esophageal tissues in tissue microarrays. C) Immunohistochemical evaluation of LTBP4 expression in normal esophagus, esophageal adenocarcinoma (EAC) stage II, EAC stage III and in a distant lymph node metastasis. Black arrows indicate LTBP4-positive staining. Data are presented ± standard deviation and *p<0.05, **p<0.01 versus normal tissue. Bar graphs: 50 µm.

Analysis of patient tissues of various adenocarcinomas of the gastrointestinal tract revealed significant downregulation of LTBP4 in comparison to normal tissues of the same organ. In detail, adenocarcinomas of esophagus showed 37%, of stomach 17%, of pancreas 9%, of small intestine 4% and of colon 22% LTBP4 expression (Figure 1A).

Furthermore, we focused more precisely on LTBP4 expression in neoplasias and preneoplasias of the esophagus (Figure 1B). Cancer adjacent normal tissue displayed 43%, hyperplasia 22%, carcinoma *in situ* 8%, EAC 37%, small cell undifferentiated carcinoma 3%, ESCC 4% and distant metastasis 4% LTBP4 expression in comparison to normal esophageal tissue. Compared to normal esophageal tissue LTBP4 expression was also significantly downregulated in patient tissue of reflux esophagitis (38%) and chronic inflammation (39%), both risk factors for EAC (Figure 1B).

Normal esophageal tissue clearly demonstrated LTBP4 expression in esophageal epithelial cells and less prominently in resident lymphocytes within the lamina propria (Figure 1C and Supporting Information S1). LTBP4 was not expressed in stage II and stage III EACs (Figure 1C). In distant lymph node metastasis, cancer cells also demonstrated no LTBP4 expression, while the surrounding mesenchymal tissue clearly showed LTBP4 expression (Figure 1C). However, no correlations between LTBP4 expression levels and clinical parameters such as pathological stage or metastatic status were found (data not shown).

### Re-expression of LTBP4 in esophageal carcinoma cell lines reduces cell migration ability

LTBP4 expression was significantly downregulated in EAC and ESCC. To investigate the role of LTBP4 in esophageal cancer more detailed, OE33, a cell line derived from EAC, and KYSE180, a cell line derived from ESCC, were analyzed.

**Figure 2:**
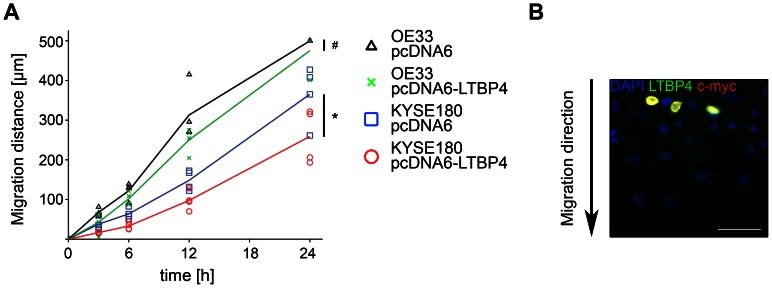
Re-expression of LTBP4 leads to less migration ability of esophageal carcinoma cells. A) Migration distance of OE33 and in KYSE180 cells after transient transfection with either pcDNA6 as a control or pcDNA6-LTBP4 within 24 h. Experiments were repeated at least 3 times. The symbols represent the results of each individual experiment and the graphs document the respective mean values. Significances are given: ^#^p = n.s. and *p<0.05. B) Representative immunofluorescence staining of the migration assay performed with KYSE180 cells re-expressing LTBP4. LTBP4 (green) and c-myc (red) positive cells (yellow) defined the borders of the cell free gap. Only non-transfected cells migrated into the cell free gap. Nuclei were counterstained with DAPI (blue). The arrow indicates the migration direction. Bar graph: 50 µm.

Both cell lines showed similar LTBP4 expression at transcriptional and protein level (Supporting Information S1). Transient re-expression of LTBP4 (transfection efficiency of 40-50%) in OE33 and KYSE180 cells resulted in a four- and nine-fold upregulation of LTBP4, respectively (Supporting Information S1). The smaller bands after transient re-expression of LTBP4 might reflect missing post-translational modifications, an effect previously described [19]. Partial proteolysis could be excluded, since LTBP4 and c-myc could be detected as a fusion protein. The transient re-expression of LTBP4 modified the ability of OE33 and KYSE180 cells to migrate. After 24 h the migration distance of OE33 cells was reduced by 5% (p = n.s.) and of KYSE180 cells by 30% (p<0.05) compared to control vector transfected cells (Figure 2A). Immunofluorescence staining revealed that LTBP4 and c-myc positive cells defined the borders of the cell free gap. Only non-transfected cells migrated into the cell free gap (Figure 2B). Cell viability and cell proliferation did not change after re-expression of LTBP4 in OE33 and KYSE180 cells (data not shown).

### Demethylation treatment with 5-aza-2’-deoxycytidine induces LTBP4 expression, whereas TGF-β1 expression is not changed

**Figure 3:**
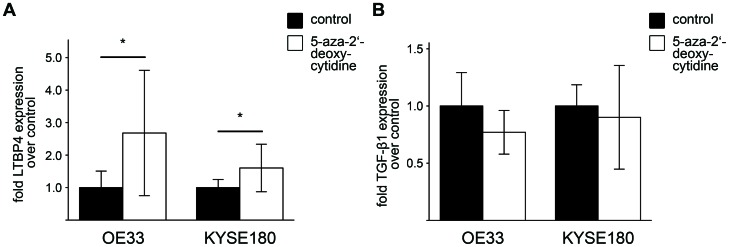
Demethylation treatment with 5-aza-2’-deoxycytidine induces LTBP4 expression, whereas TGF-β1 expression is not changed. A) qPCR analysis showed induction of LTBP4 expression in OE33 and KYSE180 cells after demethylation treatment with 5-aza-2’-deoxycytidine. B) qPCR analysis showed no significant changes in TGF-β1 expression in OE33 and KYSE180 cells after demethylation treatment with 5-aza-2’-deoxycytidine. Untreated cells served as control. Experiments were repeated at least 3 times and the mean value was calculated. Data are presented ± standard deviation and *p<0.05 versus control.

Epigenetic modifications, such as methylation of cytosine residues in CpG sequences, so called CpG islands, are known to regulate the inactivation of tumor suppressor genes in cancer cells [20]. After demethylation treatment with 5-aza-2’-deoxycytidine LTBP4 expression was induced in OE33 and KYSE180 cells by more than 150% and 60%, respectively (Figure 3A), whereas demethylation treatment with 5-aza-2’-deoxycytidine did not significantly change TGF-β1 expression in both esophageal cancer cell lines (Figure 3B).

### LTBP4 promoter regions are hypermethylated in esophageal carcinoma cell lines

**Figure 4:**
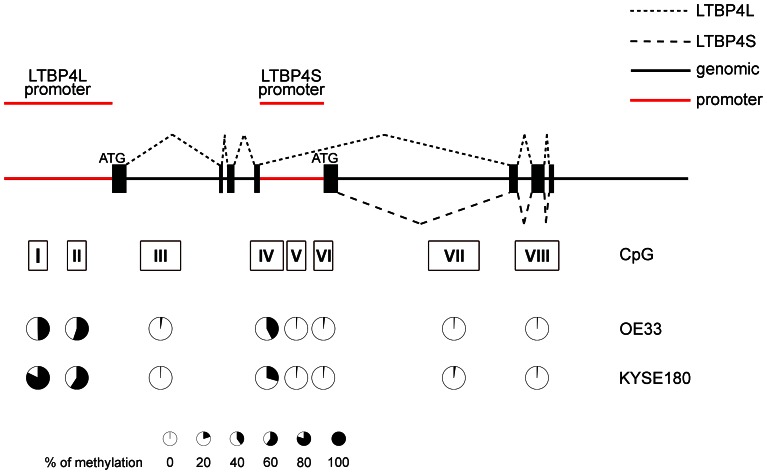
Methylation status of the two predicted LTBP4 promoter regions in esophageal carcinoma cell lines. The methylation status in the putative LTBP4 promoter regions was analyzed by clonal bisulfite sequencing in OE33 and KYSE180 cells. Eight CpG islands were identified and at least ten clones were sequenced for each cell line. The percentage of methylation in each CpG island is visualized as pie chart. Exons are illustrated by black boxes and LTBP4 promoters by red lines. The translation start sites are indicated (ATG). The dashed lines represent alternative splicing of LTBP4.

We analyzed the predicted independent promoter regions of LTBP4L and LTBP4S in the N-terminal region of the genomic *LTBP4* DNA sequence [12]. In this region eight CpG islands (I–VIII) have been found, including two CpG islands (I and II) within the predicted LTBP4L promoter and three CpG islands (IV–VI) located within the predicted LTBP4S promoter. CpG islands III, VII and VIII lie within immediate vicinity of the promoter structures (Figure 4).

Detailed analysis of the methylation status of the CpG islands in OE33 and KYSE180 revealed that in the CpG islands (III, VII, VIII) distant to the promoter regions methylation was rarely (<3%) found (Figure 4).

The CpG islands located within the LTBP4L promoter were hypermethylated (Figure 4). In OE33 and KYSE180 cells CpG island I showed an average methylation of about 50% and 80% and CpG island II 45% and 60%, respectively (Figure 4).

The CpG islands located within the LTBP4S promoter displayed different methylation patterns. CpG island V and VI showed less than 2% methylation, whereas CpG island IV showed in OE33 and KYSE180 cells an average methylation of about 40% and 30%, respectively (Figure 4).

### Identification of potential transcription factors for LTBP4L and LTBP4S

Further *in silico* analysis by internet-based search tools MatInspector [21], TFSEARCH [22] and Promoter 2.0 [23] were performed to examine whether the genomic sequence of CpG islands I, II and IV within LTBP4L and LTBP4S promoter regions contain regulatory elements. None of the common promoter elements, such as TATA- or CCAAT-boxes were found, which is consistent with previous findings [12]. It was shown that the promoter regions of LTBP4L and LTBP4S contain several XCPE sites as well as binding sites for different transcription factors [12]. CpG islands I, II, and IV did not contain XCPE sites, but putative binding sites for the transcription factors GATA1, SMAD3, E2F4, SP1, and MZF-1 were found (Supporting Information S1).

**Figure 5:**
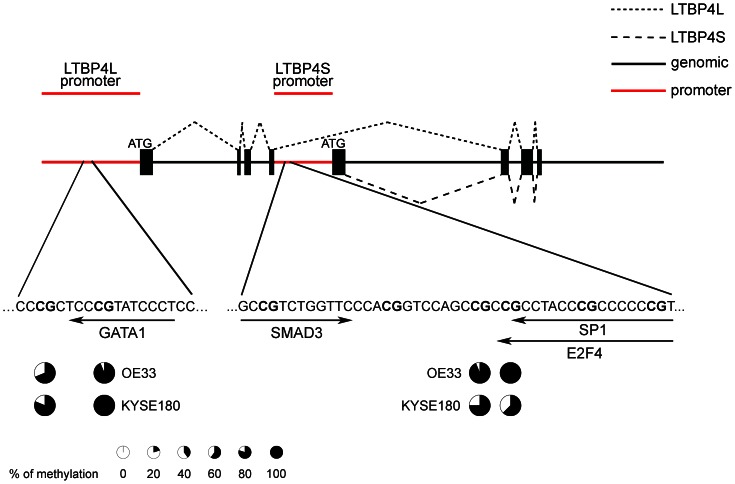
Identification of highly methylated binding sites for transcription factors in the LTBP4L and LTBP4S promoter. *In silico* analysis of LTBP4 promoter sites identified a highly methylated binding site for GATA1 in the LTBP4L promoter of OE33 and KYSE180 cells. Highly methylated binding sites for SP1 and E2F4 are found in the LTBP4S promoter of both cell lines. A binding site for SMAD3 was identified nearby. The methylation status was analyzed by clonal bisulfite sequencing and at least ten clones were sequenced for each cell line. The percentage of methylation is visualized as pie chart. Exons are illustrated by black boxes and LTBP4S and LTBP4L promoters by red lines. The translation start sites are indicated (ATG). The dashed lines represent alternative splicing of LTBP4.

Detailed examination of the methylation patterns of CpG island I revealed two highly methylated CpG dinucleotides in both cell lines as part of a putative GATA1 binding site (Figure 5).

In CpG island IV two highly methylated CpG dinucleotides were found in both cell lines (Figure 5). These CpG dinucleotides were identified as part of the putative binding site of either the transcription factor SP1 or E2F4. Additionally, 13 nucleotides upstream of the highly methylated CpG dinucleotides a binding site for SMAD3 was identified (Figure 5).

We used *in vitro* transcription factor activity studies to evaluate whether the transcription factors Gata1, SP1, SMAD3 and E2F4, identified as putative regulatory elements in CpG islands I and IV, were able to modify activity of the LTBP4L or LTBP4S promoters. The expression of the transcription factors was verified by SDS-PAGE and immunoblotting (Supporting Information S1).

**Figure 6:**
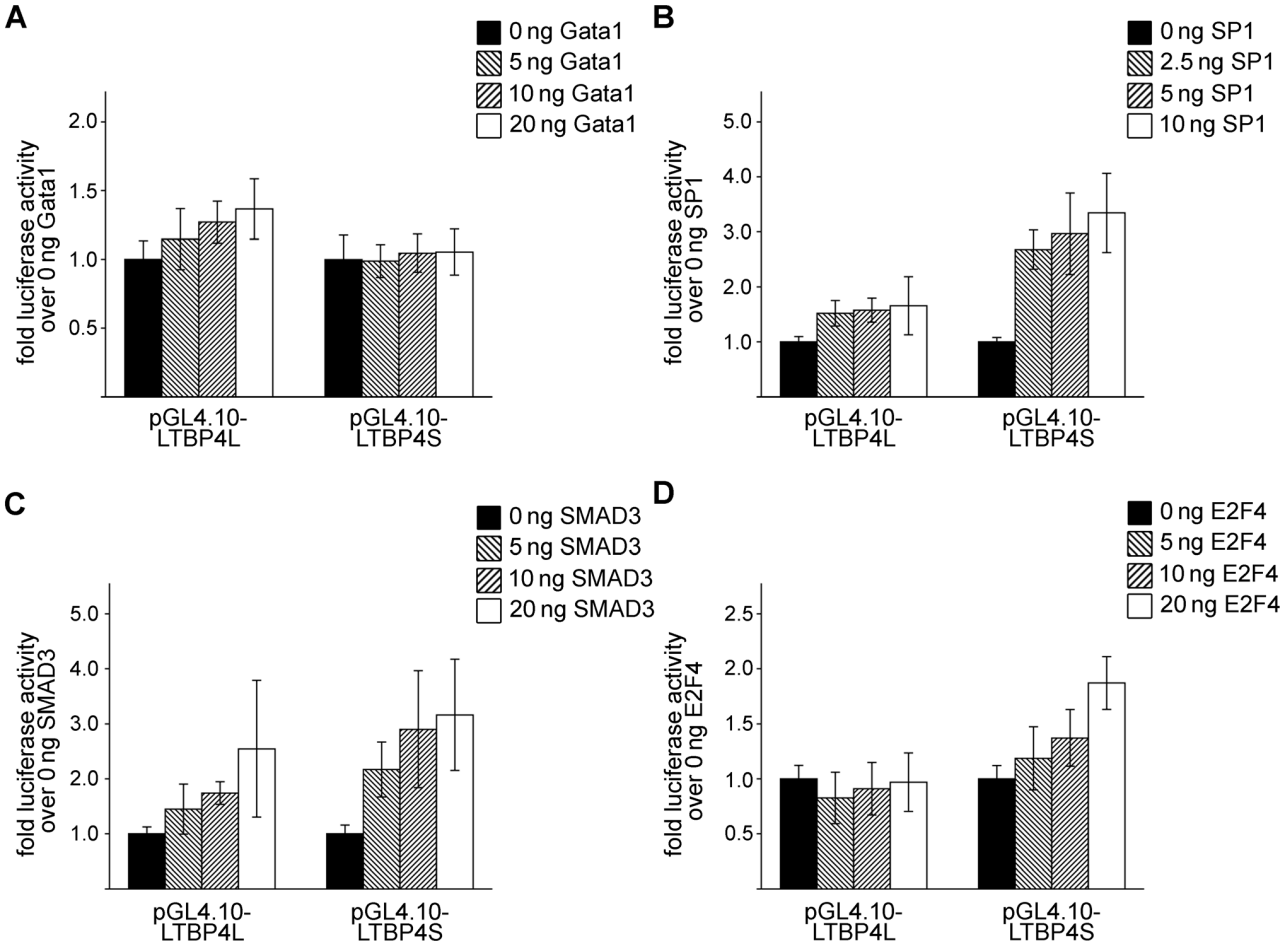
Effect of Gata1, SP1, SMAD3 and E2F4 on transcriptional activity of LTBP4L and LTBP4S promoter. HEK293 cells were transiently transfected with pGL4.10-LTBP4L or pGL4.10-LTBP4S and a *Renilla* luciferase expression vector for normalization. Cells were also co-transfected with different concentrations of A) a Gata1 expression vector, B) a SP1 expression vector, C) a SMAD3 expression vector or D) an E2F4 expression vector. After 24 h cells were lysed and luciferase activities were determined. Experiments were repeated at least 3 times and the mean value was calculated. Data are presented ± standard deviation.

As shown in Figure 6, we observed that Gata1 slightly increased LTBP4L promoter activity, but did not alter LTBP4S promoter activity (Figure 6A).

We observed a concentration dependent increase of either the LTBP4L or LTBP4S promoter activity by SP1 and SMAD3, but the LTBP4S promoter activity increase was much more prominent than the LTBP4L promoter activity increase (Figure 6B and Figure 6C). LTBP4L promoter activity was not altered by E2F4, but E2F4 slightly increased LTBP4S promoter activity (Figure 6D).

To investigate a possible enhancer function of Gata1, SP1, SMAD3 and E2F4, we also determined the activity of the LTBP4L and LTBP4S promoters linked to a minimal promoter.

**Figure 7:**
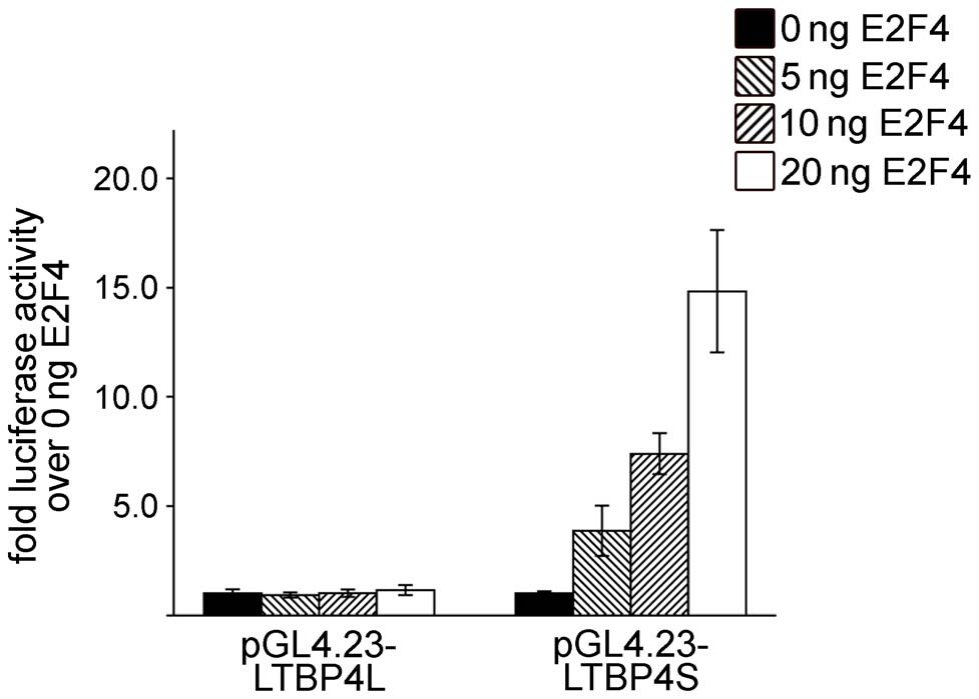
E2F4 acts as a potent regulator of LTBP4S expression in combination with other regulatory elements. HEK293 cells were transiently transfected with pGL4.23-LTBP4L or pGL4.23-LTBP4S and a *Renilla* luciferase expression vector for normalization. Cells were also co-transfected with different concentrations of an E2F4 expression vector. After 24 h cells were lysed and luciferase activities were determined. Experiments were repeated at least 3 times and the mean value was calculated. Data are presented ± standard deviation.

There were no obvious changes with Gata1, SP1, SMAD3 (data not shown) or E2F4 (Figure 7) on LTBP4L promoter activity and Gata1, SP1 or SMAD3 on LTBP4S promoter activity, when LTBP4 promoters were linked to a minimal promoter (data not shown). However, E2F4 led to a 14-fold increase of LTBP4S promoter activity, when linked to a minimal promoter (Figure 7).

## Discussion

The extracellular matrix protein latent TGF-β binding protein 4 (LTBP4) is downregulated in human and murine ductal carcinomas *in situ*, invasive mammary carcinomas [17], [24], as well as in canine mammary carcinomas [25], indicating a possible phylogenetic conserved regulation of LTBP4 expression. At the age of 6–8 months Ltbp4S knockout mice develop colorectal adenocarcinomas [26]. These results indicate that absence of LTBP4 may play a role in the development of epithelial neoplasms.

Now we found that LTBP4 is downregulated in various human adenocarcinomas of the gastrointestinal tract, as well as in neoplasias and preneoplasias of the esophagus.

To date, no molecular biomarkers for diagnosis or for risk stratification and assessment of esophageal cancer are used in clinical practice [27]. Our results suggest LTBP4 as a possible candidate gene for a biomarker panel for neoplasias and especially preneoplasias of esophageal tissue.

LTBP4 is not the only LTBP which is related to the formation of different cancer types [13], [14], [15], [16], [18], [28]. In neoplasms of liver, ovaries and neuroendocrine tumors of the digestive system expression of LTBP1 is reduced [14], [16], [18]. Ltbp3 is downregulated in tumor-specific activated mouse T cell populations in comparison to naïve T cells [28]. In nasopharyngeal carcinomas and ESCC LTBP2 expression is reduced and re-expression of LTBP2 in ESCC tumor cells suppresses neoplastic capacity *in vitro* and *in vivo* [13], [15]. Our results demonstrate a similar effect for LTBP4 *in vitro*. Re-expression of LTBP4 in EAC and ESCC cells reduces cell migration ability, whereas cell viability and cell proliferation remain unchanged. These data suggest that LTBP4 plays a specific role in promoting EAC and ESCC cell motility. However, to discover the underlying molecular mechanisms leading to reduced cell motility further investigations are required.

A well-studied transcriptional regulatory mechanism, which is often altered during malignant transformation, is the methylation of CpG islands in promoters [29]. Previously, it was shown that promoter methylation is responsible for reduced expression of LTBP2 in nasopharyngeal carcinoma and ESCC [13], [15]. Our results show that demethylation treatment leads to upregulation of LTBP4 in EAC and ESCC cell lines and higher LTBP4 expression reduces cancer cell migration. Demethylation treatment restores LTBP2 expression in ESCC cell lines and high LTBP2 expression correlates with better survival of ESCC patients [13]. Thus, epigenetic therapy of esophageal cancer with hypomethylating agents could be one new therapeutic approach, since epigenetic therapies with azacitidine demonstrate first promising results in patients with myelodysplastic syndromes, improving their quality of life and survival [30]. Of course, this novel promising therapeutic approach of esophageal cancer needs further investigations.

LTBP4L and LTBP4S are predicted to be transcribed from the control of two independent promoters [12]. Detailed analyses of methylation patterns of both promoters in cell lines derived from EAC and ESCC reveal highly methylated putative binding sites for different transcription factors, such as GATA1, SMAD3, E2F4 and SP1.

A highly methylated putative binding site for GATA1 is identified in the LTBP4L promoter region. Potential binding sites for GATA1 are also found in the upstream region of LTBP1S [31], suggesting a potential regulation of different LTBP isoforms by GATA1. However, our *in vitro* transcription factor activity studies show only a slight inducing effect for Gata1 on LTBP4L expression and no effect on LTBP4S expression.

The LTBP4S promoter region contains highly methylated putative binding sites for the transcription factors SP1 and E2F4 and additionally a putative binding site for SMAD3 is identified 13 nucleotides upstream of the highly methylated CpG dinucleotides. SP1 transcription factors are important regulators of various extracellular matrix proteins [32] and SMAD3 binding sites are necessary for induction of human LTBP3 promoter activity by TGF-β1 [33]. Our *in vitro* transcription factor activity studies show that SP1 and SMAD3 rather regulate LTBP4S than LTBP4L. Furthermore, E2F4 shows the most prominent effect on the regulation of LTBP4S, but E2F4 acts only as a potent regulator of LTBP4S expression in combination with a TATA-box as regulatory element. To our knowledge, these results are the first to reveal a connection between LTBPs and the transcription factor E2F4. Previous studies identified an important role for E2F4 in the regulation of differentiation and development of various cells, such as adipocytes [34], erythrocytes [35], [36] and calvarial osteoblast progenitor cells [37]. Deficiency of E2f4 in mice leads to postnatal death due to an impaired development of the airway epithelium [38]. Interestingly, humans with mutations in LTBP4 show a similar, slightly milder lung phenotype, die in early life due to an impaired pulmonary development and display craniofacial aberrations, including microretrognathia, flat midface, receding forehead and wide fontanelles [39]. Ltbp4S knockout mice also develop a pulmonary phenotype with impaired pulmonary development, but no obvious craniofacial aberrations [26], [40]. These results are promising indicators for a regulatory effect of E2F4 on LTBP4S expression, with respect to pulmonary development. This interesting new aspect needs further investigations.

We now show that LTBP4 is downregulated in EAC and ESCC via promoter methylation, which suggest LTBP4 as a potential prognostic biomarker that may enrich therapeutic options. Additionally, we identified the transcription factor E2F4 as new powerful regulator for LTBP4S expression.

## Materials and Methods

### Tissue microarrays, immunohistochemical staining and scoring

Tissue microarrays (TMAs) for esophageal cancer progression, multiple stomach cancer, pancreatic cancer, small intestine cancer and colon carcinoma each including additional tissue samples of normal squamous mucosa and submucosa of the esopagus, normal gastric cardia, normal pancreas, normal small intestine epithelial cells and mucosa and normal colon were purchased from US Biomax (USA). The TMA sections were stained with a primary antibody raised against an N-terminal fragment of LTBP4 (Supporting Information S1). Antigen retrieval was done by microwaving in citrate buffer (pH 6.0) for 10 minutes. Primary antibody was diluted at 1∶100 and incubated at room temperature for 1 h. The antibody was detected using the SuperVision RED 2 AP-polymer kit according to manufacturer’s protocol (DCS Innovative Diagnostik-Systeme, Germany). The TMA sections were counterstained using hematoxylin, dehydrated, and coverslipped. For positive immunoreactivity, grading was done semiquantitatively on a five-tier scale where 0 = less than 10%, 1 = 10–25%, 2 = 25–50%, 3 = 50–75%, 4 = more than 75% positive for LTBP4 staining. No attempt was made to grade results based on staining colour intensity, because of the inherent subjectivity of such a measurement, and its susceptibility to variations between investigators. The evaluation was performed independently by two experienced investigators who were unaware of the related clinical information and the conflicting scores were resolved at a discussion microscope.

### Cell lines

OE33, a cell line derived from adenocarcinoma of the esophagus, was purchased from the European Collection of Cell Cultures (ECACC, UK). KYSE180, a cell line derived from esophageal squamous cell carcinoma, was purchased from the German Collection of Microorganism and Cell Cultures (DSMZ, Germany). All esophageal cell lines were cultured in RPMI 1640 supplemented with 10% fetal calf serum and 1% 10.0000 U/ml penicillin/10.000 µg/ml streptomycin at 37°C and 5% CO_2_.

### Transfection and migration assay

Human cDNA of LTBP4L (NM_001042544) and LTBP4S (NM_001042545) were purchased from OriGene (USA) and cloned (primers in Supporting Information S1) into a pcDNA6/*myc-*His vector (Life Technologies, Germany). OE33 and KYSE180 cells were seeded at 1,5×10^4^/well and 2×10^4^/well, respectively, in Culture-Inserts for migration assays (ibidi, Germany) and were transiently transfected with 1∶1 mixture of pcDNA6/*myc*-His expression vectors for LTBP4L and LTBP4S 24 h later. The Culture-Inserts were removed 18 h after transfection. The migrated distance of the cells was measured at 0 h, 3 h, 6 h, 12 h and 24 h after removal of the Culture-Inserts. The transfection efficiency was determined by immunofluorescence and the protein expression by SDS-PAGE and immunoblotting.

### Immunofluorescence

Cells were fixed 24 h after removal of the Culture-Inserts for 15 minutes in 4% paraformaldehyde and blocked for 60 minutes in 5 % bovine serum albumin/0.3% Triton X-100 in PBS. Cells were incubated with primary antibodies (Supporting Information S1) over night at 4°C. Secondary antibodies were coupled to Alexa Fluor 488 or Cy3 (Life Technologies, Germany). Nuclei were counterstained with DAPI (Life Technologies, Germany).

### RNA Expression Analysis

Confluent cultures of the esophageal cells were used for the extraction of RNA with the TRIzol reagent (PEQLAB Biotechnologie, Germany) according to manufacturer’s instruction. RNA concentrations and purities were determined spectrophotometrically. Reverse transcription was performed with oligo dT primers and SuperScript III Reverse Transcriptase (Life Technologies, Germany) using 1 µg total RNA according to manufacturer’s instructions. The obtained cDNA samples were used for quantitative real-time PCR (qPCR) analysis. qPCR was done using the Platinum® Quantitative PCR SuperMix-UDG w/ROX (Life Technologies, Germany). Triplicate reactions were set up and qPCR products were verified by agarose gel electrophoresis. LTBP4 and TGF-β1 quantification of mRNA levels was calculated using the standard curve method. Standard curves were created using ten-fold dilutions of an external control. Used primers and probes (Eurofins MWG Operon, Germany) are listed in Supporting Information S1.

### SDS-PAGE and Immunoblotting

Confluent cultures of the esophageal cells were harvested in RIPA buffer (50 mM Tris-HCl, pH 7.5; 150 mM NaCl; 1% NP-40; 0,5% Na-deoxycholate; 0,1% SDS), supplemented with protease inhibitor cocktail (Roche, Switzerland). Protein concentrations were determined colorimetrically by Bradford assay using the Protein Assay Kit (Biorad, USA). 100 µg of total cell proteins were separated by SDS-PAGE (7.5% polyacrylamide gels) and transferred onto nitrocellulose membranes (Macherey-Nagel, Germany). Following blocking with 5% milk powder or 5% BSA in TBST, primary antibodies (Supporting Information S1) were used for overnight incubation of nitrocellulose membranes. Glyceraldehyde-3-phosphate dehydrogenase (GAPDH) served as loading control. The antigens were detected using peroxidase-conjugated anti-rabbit- or anti-mouse-antibody (Jackson ImmunoResearch, USA). Chemiluminescence signal were measured using ImageLab measurement software (Biorad, USA).

### Methylation analysis

Genomic DNA from esophageal cancer cell lines were extracted by standard procedures and modified using the EpiTect Bisulfite Kit (QIAGEN, Germany) according to the manufacturer’s protocol. Two promoter regions of LTBP4 were identified previously [12]. Using the UCSC Genome Browser [41] eight CpG islands (CG content >50%) were identified within the promoter regions. Methylation specific primers were designed with MethPrimer [42]. Used primers are listed in Supporting Information S1. The amplified bisulfite-treated CpG islands were then subcloned into the pGM-T Easy Vector (Promega, USA). At least ten clones of each esophageal cancer cell line were analyzed.

The esophageal cancer cell lines were treated with 5 µM 5-aza-2’-deoxycytidine (Sigma-Aldrich, Germany) for 5 days to determine the significances of hypermethylation in *LTBP4* silencing.

### Luciferase reporter gene assay

The promoter regions of LTBP4L and LTBP4S were amplified using specific primers containing restriction sites for *Bgl*II and *Hin*dIII (Supporting Information S1), respectively. These fragments were cloned into pGL4.23 and pGL4.10 vectors (Promega, USA), which results in pGL4.23-LTBP4L, pGL4.23-LTBP4S, pGL4.10-LTBP4L and pGL4.10-LTBP4S.

HEK293 cells were plated on 96-well plates, grown to a density of 30–50% and transfected with the LTBP4 promoter constructs or empty pGL4.23 and pGL4.10 vectors and the *Renilla* luciferase expression vector pGL4.74 (Promega, USA) using jetPRIME (PEQLAB Biotechnologie, Germany). The cells were cotransfected with expression vectors for murine Gata1 (Addgene plasmid 13626; [43], [44]), human SP1, human SMAD3 (Addgene plasmid 10920; [45]) or human E2F4 (Addgene plasmid 10914; [46]). 24 h after transfection, luciferase was measured in triplicate using the Beetle-Juice BIG KIT (P.J.K., Germany) and normalized for *Renilla* luciferase activity (Renilla-Juice BIG KIT, P.J.K., Germany). To verify the expression of the transcription factors in HEK293 cells SDS-PAGE and subsequent immunoblotting was performed (Supporting Information S1).

### Statistical evaluation

Unless otherwise indicated, data always reflect mean values ± standard deviation. To assess statistical significance One-way Analysis of Variance (1-way ANOVA) followed by post-test analysis with Bonferroni correction or two-tailed Student’s t-test were used (^#^p = n.s, *p<0.05, **p<0.01). Calculations were performed using SPSS (IBM, Germany).

## Supporting Information

Supporting Information S1 **Supporting Tables and Figures.**
(PDF)
