# Supplementary material for: Latent Transforming Growth Factor β-Binding Protein 4 Is Downregulated in Esophageal Cancer via Promoter Methylation
Source: PLoS One. 2013 May 31;8(5):e65614. doi: 10.1371/journal.pone.0065614 (PMC3669142; doi:10.1371/journal.pone.0065614)
Supplement: Supporting Information S1 — Supporting Tables and Figures. (PDF) [file pone.0065614.s001.pdf]

Latent transforming growth factor β-binding protein 4 is downregulated in esophageal cancer via promoter methylation

Insa Bultmann[1*], Anne Conradi[1*], Celine Kretschmer[2], Anja Sterner-Kock[1#]

[1]Center for Experimental Medicine, Medical Faculty, University of Cologne, Cologne, Germany

[2]Med. Klinik Hepatologie & Gastroenterologie, Charité Berlin, Berlin, Germany.

* These authors contributed equally to this work.

[#] E-mail: anja.sterner-kock@uk-koeln.de

Supporting Information S1. Supporting Tables and Figures.

Table S1. Used antibodies.
(Material and Methods: Tissue microarrays, immunohistochemical staining and scoring; Immunofluorescence; SDS-PAGE and Immunoblotting)

Table S2. Used PCR primers.
(Material and Methods: Methylation analysis; Luciferase reporter gene assay; Transfection and migration assay)

Table S3. Used qPCR primers.
(Material and Methods: RNA Expression Analysis)

Figure S1. LTBP4 expression in normal esophageal tissue.
(Results: LTBP4 is downregulated in different stages of esophageal cancer progression)

Figure S2. LTBP4 expression in OE33 and KYSE180 cells before and after re-expression of LTBP4.
(Results: Re-expression of LTBP4 in esophageal carcinoma cell lines reduces cell migration ability)

Figure S3. Highly methylated CpGs and potential transcription factor binding sites in CpG island I.
(Results: Identification of potential transcription factors for LTBP4L and LTBP4S)

Figure S4. Highly methylated CpGs and potential transcription factor binding sites in CpG island II.
(Results: Identification of potential transcription factors for LTBP4L and LTBP4S)

Figure S5. Highly methylated CpGs and potential transcription factor binding sites in CpG island IV.
(Results: Identification of potential transcription factors for LTBP4L and LTBP4S)

Figure S6. Expression analysis of Gata1, SP1, SMAD3 and E2F4 after transfection with expression vectors.
(Results: Identification of potential transcription factors for LTBP4L and LTBP4S; Material and Methods: Luciferase reporter gene assay)

Table S1. Used antibodies.

LTBP4 – Immunohistochemical staining	H-293, Santa Cruz Biotechnologies, USA
LTBP4 – Western Blot	GTX101725, GeneTex, USA
GAPDH	#2118, Cell Signaling, USA
c-Myc	IMG-6824A, Biomol, Germany
Gata1	#3535, Cell Signaling, USA
SP1	#9389, Cell Signaling, USA
E2F4	ABIN483876 , antikoerper-online.de, Germany
SMAD3	#9523, Cell Signaling, USA

Table S2. Used PCR primers.

LTBP4L promoter	for (*Bgl*II)	AAG ATC TGA ACT CCT GAC CTC ATG ATC
	rev (*Hin*dIII)	CTA AGC TTG AAG AGC GGC AGC AAC AG
LTBP4S promoter	for (*Bgl*II)	AAG ATC TCG TTG TAG CTC AGC ACC CA
	rev (*Hin*dIII)	CTA AGC TTC AGC AGC ACC AAT AGC GAC
LTBP4 cloning primers	for (*Bam*HI)	AGT GCG GGA TCC TGG ACG
	rev (*Xba*I)	GCA ATC TAG AGT CGA GTC GGG CCC
CpG I -methylation specific	for	GTG AAT TTG GGA TTT TTA GAG G
	rev	CAA TCA AAA AAC AAC AAA AAC AAA T
CpG II -methylation specific	for	TTG TTG GTG TTG TTG TTG
	rev	CCC TAC CCC AAT AAA CTA AA
CpG III -methylation specific	for	CCT ATA GAT CTG GGG GCT ATA GG
	rev	GAG GCT CCC CAC CAG ATG GCC TCA T
CpG IV -methylation specific	for	GAT TAT TGA GAA GGA GGT TTT TAT AG
	rev	CCC CAC AAT CTT AAA CAT TTA ATA C
CpG V -methylation specific	for	TAT TAT TTT TAA TGG ATT TTT TTT TT
	rev	CCC CCT CCT CAC CAC TCC
CpG VI -methylation specific	for	GTT AGG GTT GTA GTT TGG ATT GG
	rev	AAA ATC TAA ACT CCC TCT CCC TAA A
CpG VII -methylation specific	for	TTG TTT TTA GTT GTT TTT TGT AGT TT
	rev	CCC CTA ACT ATA CCC CCT TTA TAA C
CpG VIII -methylation specific	for	GGT AAG TTT TGT TAG TTG TAT TTT T
	rev	ATA AAA TTA TCA CCA CTC CCC AAC

Table S3. Used qPCR primers.

LTBP4	for	GCT GCC CTG TGT GAA AAT GTC
	rev	GGG AAC GTG CCA GCA GAA
	probe	AAC AGC CCG GAA GAG TTT GAC CCC A
LTBP4 - external control	for	GGC TGG AGT GCG TTG ATA AT
	rev	AGG GGT CGT AGG GTA GCA CT
TGF-ß1	for	GTA CCT GAA CCC GTG TTG CT
	rev	GTA TCG CCA GGA ATT GTT GC
	probe	TAA AAG TGG AGC AGC ACG TG
TGF-ß1 - external control	for	TCG CCC TGT ACA ACA GCA
	rev	GAA CCC GTT GAT GTC CAC TT

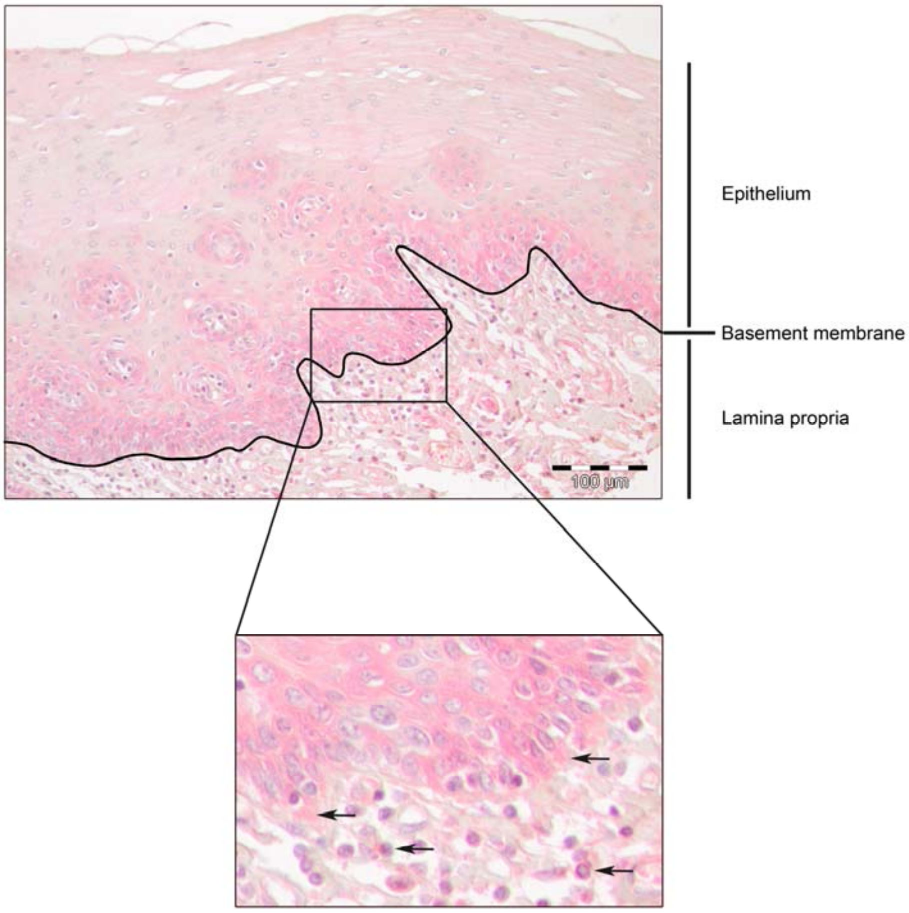

Figure S1. LTBP4 expression in normal esophageal tissue.

Immunohistochemical evaluation of LTBP4 expression (red immunoreactivity) in normal esophageal tissue. Normal esophageal tissue clearly demonstrated LTBP4 expression in esophageal epithelial cells and less prominently in resident lymphocytes within the lamina propria (black arrows). Bar graph: 100 µm.

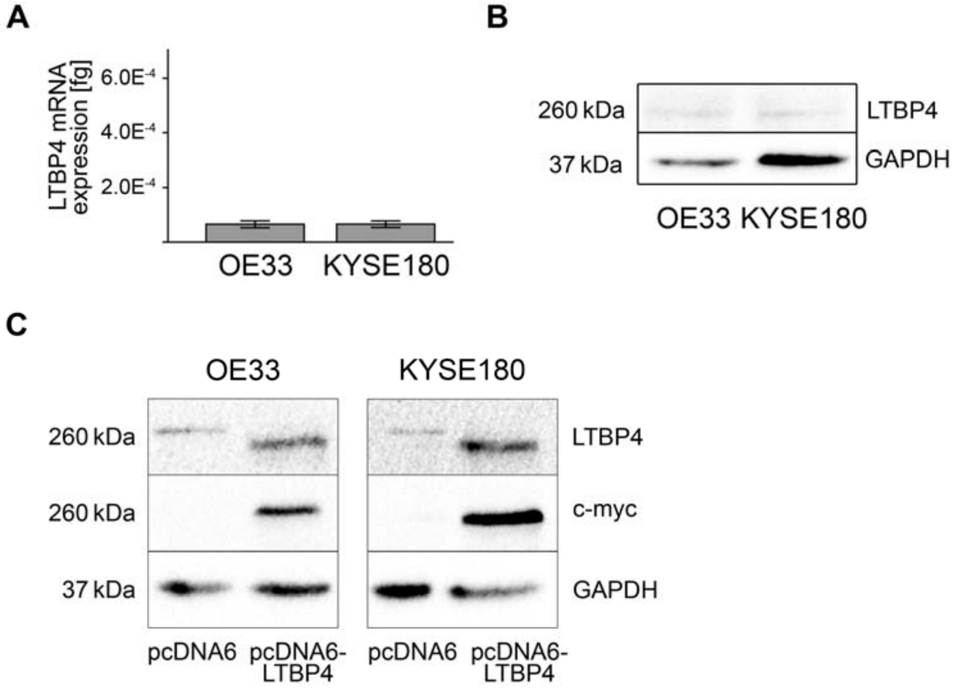

Figure S2. LTBP4 expression in OE33 and KYSE180 cells before and after re-expression of LTBP4.

A) Amount of LTBP4 mRNA measured in fg in the esophageal adenocarcinoma cell line OE33 and the esophageal squamous cell carcinoma cell line KYSE180. The amount of mRNA was assessed by qPCR. B) Western Blot analysis of LTBP4 and GAPDH in OE33 and KYSE180 cells. C) Western Blot analysis of LTBP4, c-myc and GAPDH in OE33 and KYSE180 cells after pcDNA6 as a control or pcDNA6-LTBP4 transient transfection.

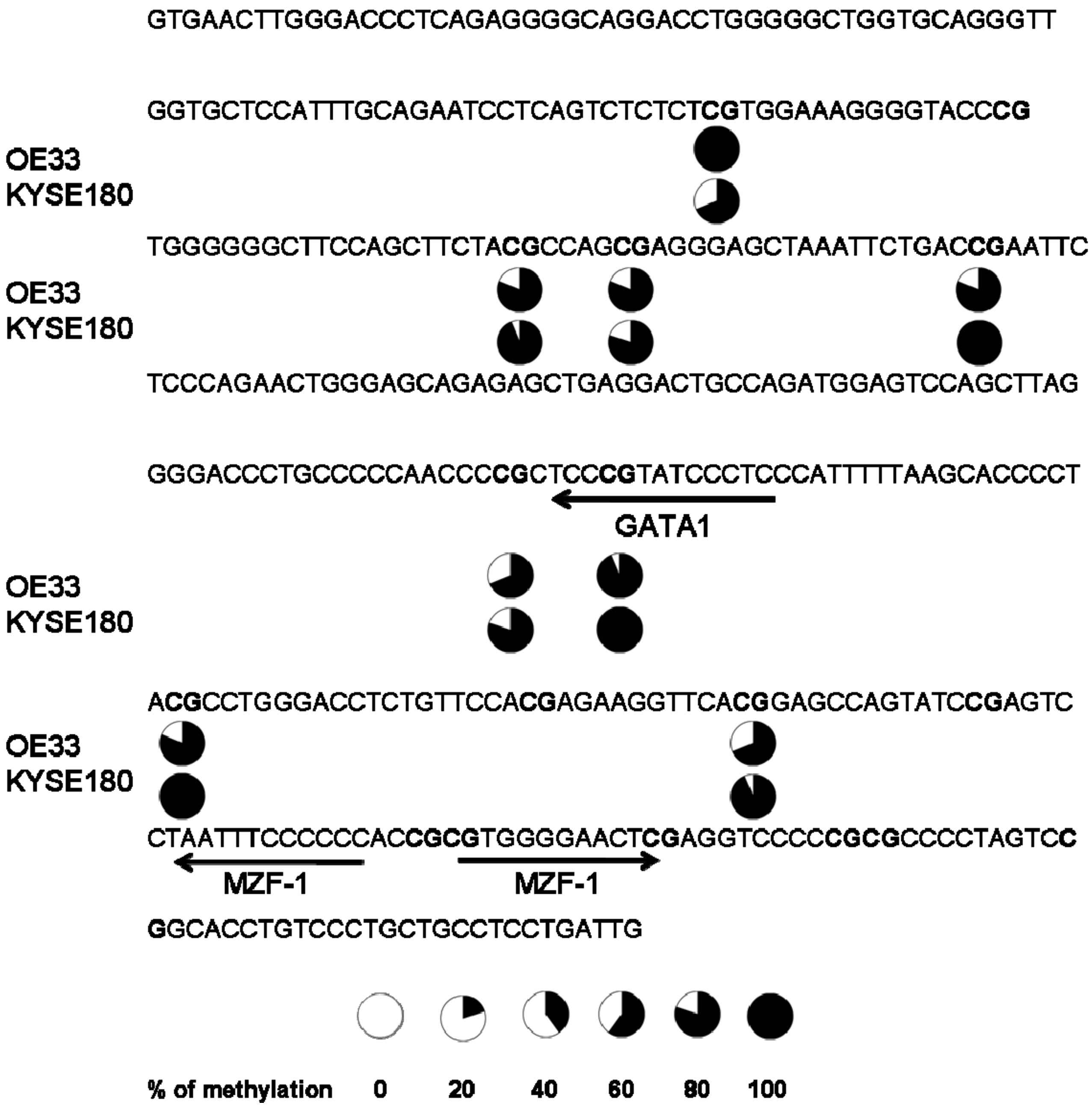

Figure S3. Highly methylated CpGs and potential transcription factor binding sites in CpG island I.

In silico analysis of the DNA sequence of CpG island I within the putative LTBP4L promoter identified a highly methylated binding site for GATA1 in OE33 and KYSE180 cells. Two putative binding sites for MZF-1 were not methylated. The methylation status was analyzed by clonal bisulfite sequencing and at least ten clones were sequenced for each cell line. The percentage of methylation is visualized as pie chart.

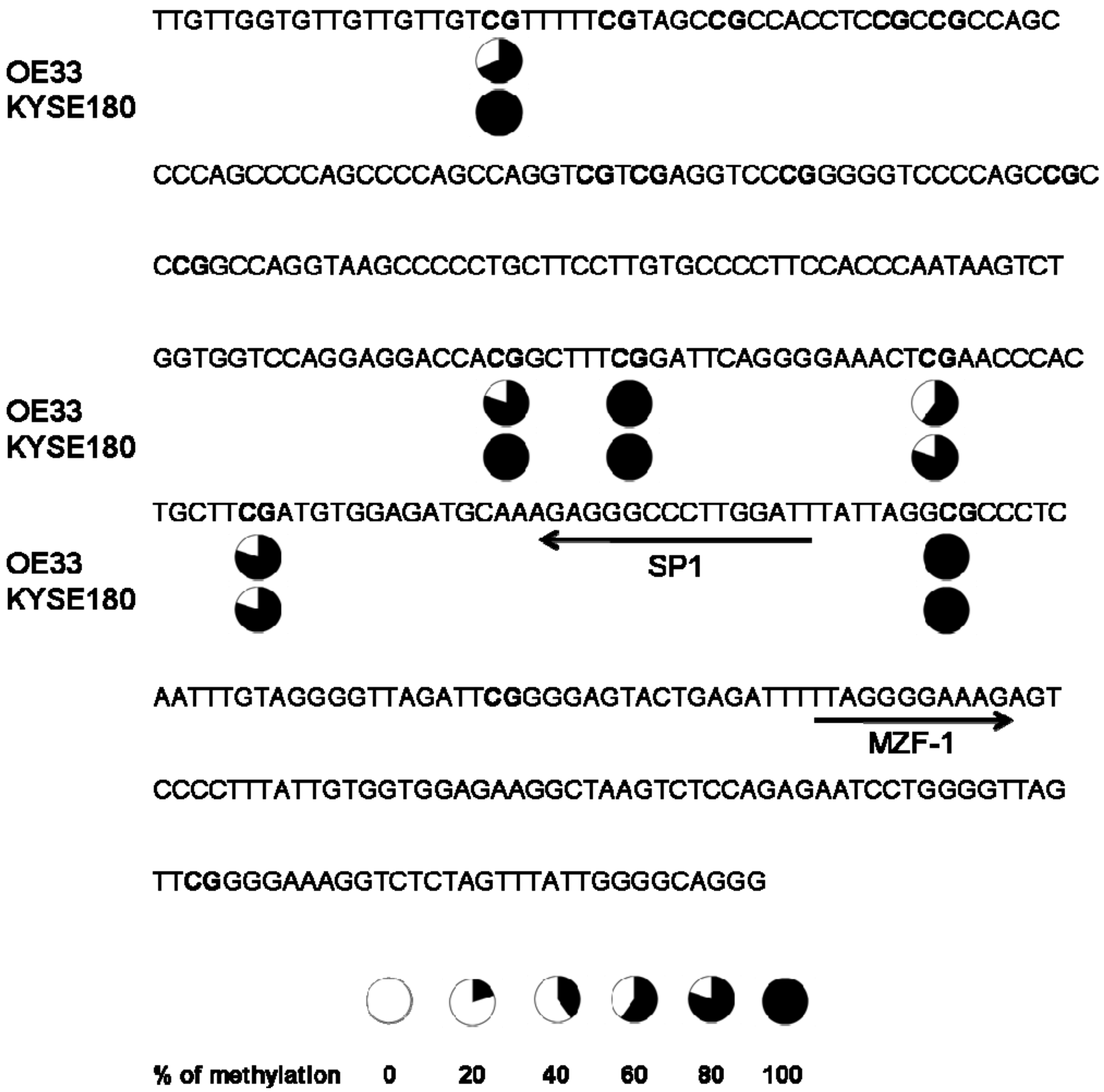

Figure S4. Highly methylated CpGs and potential transcription factor binding sites in CpG island II.

In silico analysis of the DNA sequence of CpG island II within the putative LTBP4L promoter identified not methylated binding sites for SP1 and MZF1 in OE33 and KYSE180 cells. The methylation status was analyzed by clonal bisulfite sequencing and at least ten clones were sequenced for each cell line. The percentage of methylation is visualized as pie chart.

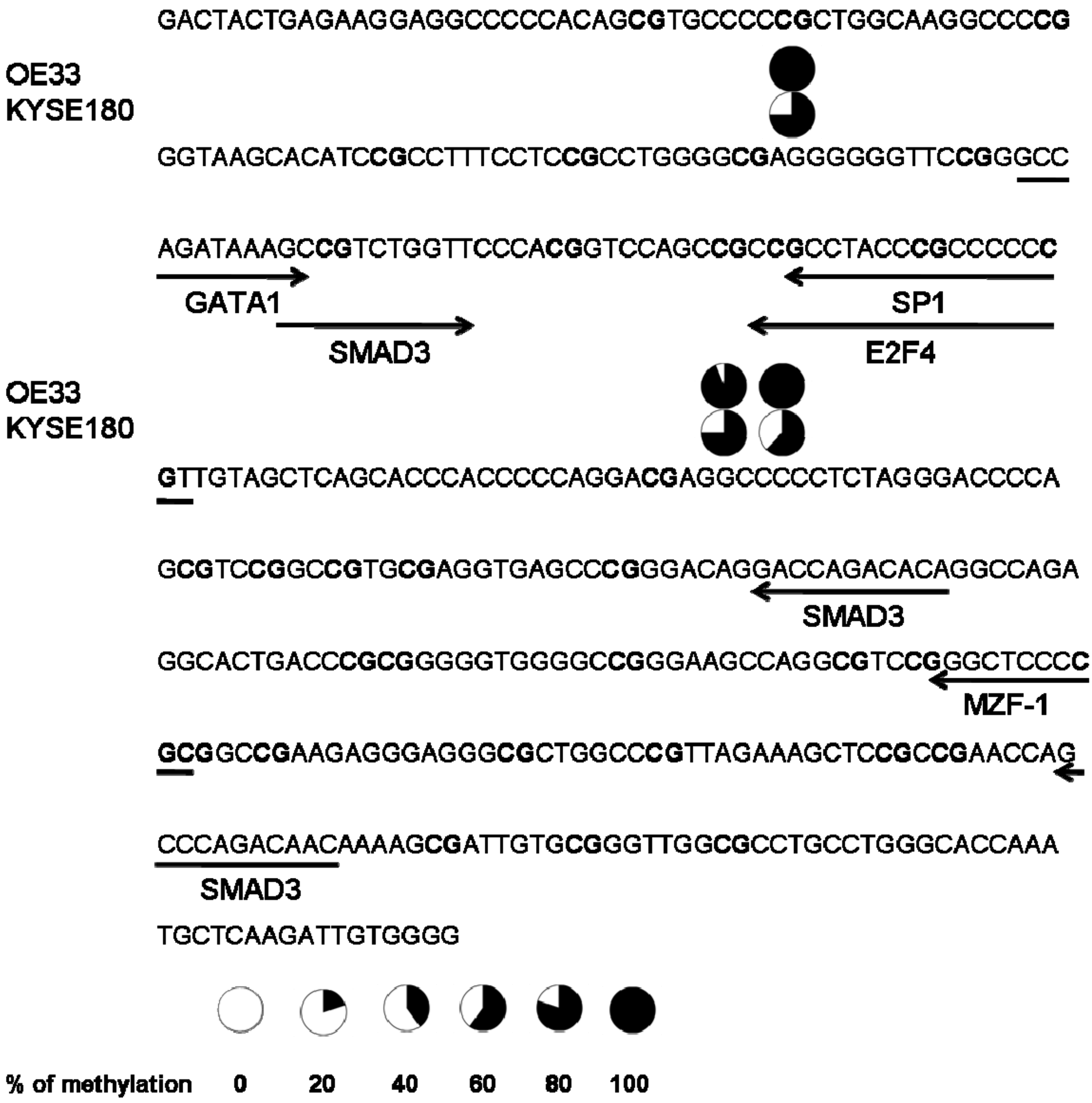

Figure S5. Highly methylated CpGs and potential transcription factor binding sites in CpG island IV.

In silico analysis of the DNA sequence of CpG island IV within the putative LTBP4S promoter identified two highly methylated binding sites for SP1 and E2F4 in OE33 and KYSE180 cells. Binding sites for GATA1, SMAD3 and MZF-1 were not methylated. The methylation status was analyzed by clonal bisulfite sequencing and at least ten clones were sequenced for each cell line. The percentage of methylation is visualized as pie chart.

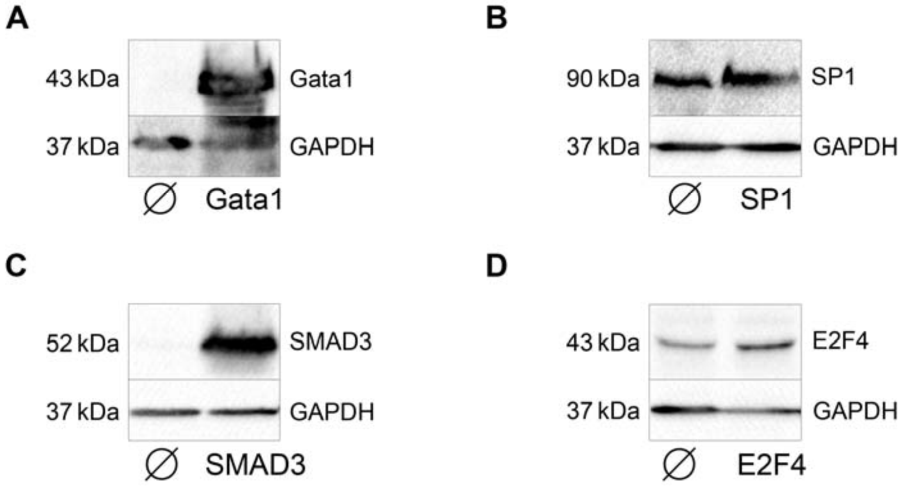

Figure S6. Expression analysis of Gata1, SP1, SMAD3 and E2F4 after transfection with expression vectors.

Western Blot analysis of HEK293 cells after transfection with A) a Gata1 expression vector, B) a SP1 expression vector, C) a SMAD3 expression vector or D) an E2F4 expression vector in comparison to non-transfected cells (Ø).
